# Guilt Facets and Eating Disorder Psychopathology: An Exploratory Cross-Sectional Study Using the Moral Orientation Guilt Scale

**DOI:** 10.3390/medicina62091719

**Published:** 2026-09-07

**Authors:** Fabiola Raffone, Eugenia Barone, Danilo Atripaldi, Eleonora Arsenio, Flavia Martinelli, Serena Cruccu, Marco Carfagno, Vassilis Martiadis, Alessio Maria Monteleone

**Affiliations:** 1 Department of Psychiatry, University of Campania “L. Vanvitelli”, 80138 Naples, Italy; 2 Department of Mental Health, Asl Napoli 1 Centro, 80145 Naples, Italy; 3 Department of Advanced Medical and Surgical Sciences, University of Campania “L. Vanvitelli”, 80138 Naples, Italy

**Keywords:** moral emotions, guilt, eating disorders, binge-eating disorder, bulimia nervosa, anorexia nervosa, asceticism, Moral Orientation Guilt Scale, EDI-2

## Abstract

*Background and Objectives:* Moral emotions are increasingly implicated in the maintenance of eating disorders (EDs), yet guilt is less differentiated than shame in the ED literature. Contemporary cognitive accounts distinguish deontological guilt linked to violating internalised moral norms from altruistic guilt linked to empathic concern or perceived harm to others. This study examined associations between guilt facets, ED diagnosis, and ED psychopathology in a clinical sample. *Materials and Methods:* One hundred and eleven adult outpatients (≥18 years) with DSM-5 ED diagnoses (anorexia nervosa, *n* = 32; bulimia nervosa, *n* = 24; binge-eating disorder, *n* = 55; 92 women) were consecutively recruited at first assessment at the Eating Disorders Centre of the University of Campania “Luigi Vanvitelli” (Naples, Italy) between January 2024 and December 2025 and completed the Italian versions of the Moral Orientation Guilt Scale (MOGS) and the Eating Disorder Inventory 2 (EDI-2). Between-diagnosis differences in guilt facets were tested using Kruskal–Wallis analyses. Spearman correlations examined relationships between MOGS facets and EDI-2 subscales. An exploratory regression tested whether MOGS Empathy was associated with EDI-2 Asceticism beyond sex. *Results:* Significant between-diagnosis differences emerged for Moral Norm Violation (*p* < 0.001), Moral Dirtiness (*p* = 0.020), Harm (*p* = 0.021), and both deontological (*p* = 0.001) and altruistic guilt composites (*p* = 0.041), with binge-eating disorder showing the highest mean levels. No between-diagnosis difference emerged for Empathy (*p* = 0.114). The prespecified MNV–Bulimia correlation was not confirmed (ρ = 0.12, unadjusted *p* = 0.199; Holm-adjusted *p* = 0.199). Empathy was associated with Asceticism (ρ = 0.28, unadjusted *p* = 0.003; Holm-adjusted *p* = 0.008), and altruistic guilt was associated with Interpersonal Distrust (ρ = 0.24, unadjusted *p* = 0.010; Holm-adjusted *p* = 0.020). Broader exploratory correlations did not survive false discovery rate correction. In a regression model including sex, Empathy was associated with Asceticism (B = 0.263, 95% CI 0.078–0.447; standardised β = 0.26; *p* = 0.006), explaining approximately 7% of the variance (R^2^ = 0.07). *Conclusions:* Distinct guilt facets show selective, small-to-moderate associations with ED-related psychological traits in an adult outpatient sample. These findings are hypothesis-generating and require replication in larger and longitudinal samples that include shame, trauma, self-criticism, weight stigma, illness duration, and gender identity measures.

## 1. Introduction

Eating disorders (EDs) are severe psychiatric conditions characterised by persistent disturbances in eating behaviour and weight/shape evaluation [1,2], with substantial medical morbidity and elevated all-cause and cause-specific mortality, particularly in anorexia nervosa [3,4,5].

Transdiagnostic cognitive–behavioural models emphasise dietary restraint, rigid self-evaluative standards, and maladaptive affect regulation as maintaining mechanisms across ED presentations, providing the basis for enhanced cognitive behaviour therapy (CBT-E) and related interventions [6,7]. A recent systematic review supports the efficacy and effectiveness of CBT-E across a range of ED diagnoses, although comparative superiority over other active treatments remains less consistent [8]. A meta-analytic literature on risk and maintenance factors also supports the relevance of negative affect and self-evaluative processes in the emergence and persistence of eating pathology [9].

Theories of affect regulation in EDs have increasingly incorporated moral emotions, especially shame and guilt. Although both are self-evaluative emotions, shame typically involves global devaluation of the self (“I am bad”), whereas guilt is linked to appraisals of a specific behaviour or its consequences (“I did something bad”), and may motivate reparative or avoidance strategies depending on context and intensity [10]. Across psychopathology, shame is more consistently linked to maladjustment than guilt, while guilt shows more mixed associations that may depend on the type of guilt assessed [11].

In ED research, shame has received sustained attention and shows robust cross-sectional associations with ED symptom severity [12]. In contrast, guilt is less consistently differentiated, and reviews of clinical ED samples have highlighted substantial heterogeneity in how guilt is measured, conceptualised, and linked to specific symptoms [13]. A related body of evidence in binge eating has also emphasised the roles of shame and self-disgust, but suggests that guilt may be a frequent co-occurring moral affect state around episodes of loss of control eating [14]. Using the Moral Orientation Guilt Scale (MOGS), our recent pilot study in an outpatient ED sample suggested that deontological guilt facets were associated with binge/purge features, whereas altruistic guilt was associated with interpersonal distrust [15].

Several ecological momentary assessment (EMA) studies provide temporal support for guilt-related processes in dysregulated eating. In bulimia nervosa, guilt increases sharply around binge-only, purge-only, and binge/purge events [16], and guilt decreases after binge-eating episodes in ways that may vary by diagnosis and compensatory behaviour [17]. In adults with higher body weight, guilt and other negative affects also show systematic fluctuations before and after binge-eating episodes [18]. In binge-eating disorder, guilt has been identified as a prominent affective state that tends to increase prior to binge eating and decrease afterwards, consistent with an affect-regulation cycle [19]. Moreover, in bulimia-spectrum samples, daily and momentary negative affect, including guilt, is closely coupled with bulimic behaviours over time [20].

Importantly, guilt-relevant affective dynamics are not limited to binge eating. In anorexia nervosa, restrictive eating may be reinforced by short-term reductions in aversive affect, including guilt, in the natural environment [21]. In parallel, research in higher-weight populations suggests that weight-related shame and guilt are associated with binge-eating severity [22], and that subjective “feeling fat” experiences may activate moral emotions (disgust, guilt, shame) that, in turn, contribute to objective binge episodes at both between- and within-person levels [23]. Together, these findings support the hypothesis that guilt acts as both a state and a trait-like process that interacts with ED behaviours.

However, contemporary cognitive accounts suggest that guilt is not a unitary construct. Deontological guilt is linked to violating internalised moral rules (e.g., contamination/moral dirtiness), whereas altruistic guilt is linked to perceived harm to others, empathic concern, or unfair advantage [24]. The Moral Orientation Guilt Scale (MOGS) operationalises these orientations through four facets—Moral Norm Violation (MNV), Moral Dirtiness (MODI), Empathy, and Harm—and allows for the computation of deontological and altruistic composite indices [25]. While informative, our pilot work was limited by its sample size and requires replication with an expanded dataset [15].

Building on our pilot work [15], the present cross-sectional study analysed an expanded outpatient clinical sample to examine whether guilt differs in its components among individuals with anorexia nervosa, bulimia nervosa, and binge-eating disorder and to explore associations between guilt facets and ED psychopathology as measured by the Eating Disorder Inventory 2 (EDI-2). Consistent with theory [24,25] and prior findings [15,16,17,18,19,20], we expected deontological guilt facets to relate to bulimic symptomatology and empathy-related guilt to relate to asceticism. Building on the empathy–asceticism finding from our pilot study [15], we additionally tested, in an exploratory regression model, whether MOGS Empathy was associated with EDI-2 Asceticism after accounting for sex and, in a sensitivity model, for age, BMI, and diagnosis. All remaining tests were treated as exploratory and interpreted cautiously.

## 2. Materials and Methods

### 2.1. Study Design and Setting

This exploratory cross-sectional study was conducted at the Eating Disorders Centre of the University of Campania “Luigi Vanvitelli” (Naples, Italy). Consecutive patients were recruited at first clinical assessment and completed study questionnaires prior to treatment initiation. The study was conducted in accordance with the Declaration of Helsinki and approved by the Ethics Committee of the University of Campania “Luigi Vanvitelli” (protocol code 09.32-20210013912, approved on 3 September 2021). All participants provided written informed consent.

### 2.2. Participants

The analytic sample comprised 111 adults (≥18 years) with DSM-5 diagnoses of anorexia nervosa (AN, *n* = 32), bulimia nervosa (BN, *n* = 24), or binge-eating disorder (BED, *n* = 55). Ninety-two participants were women. The 43 participants from our previously published pilot study are included in this expanded sample; therefore, comparisons with the pilot should be interpreted as a partial internal replication in an expanded cohort rather than as an independent replication.

Participants were consecutively recruited between January 2024 and December 2025 at the Eating Disorders Centre of the University of Campania “Luigi Vanvitelli”. Eligibility criteria were: (i) age ≥ 18 years; (ii) a DSM-5 diagnosis of anorexia nervosa, bulimia nervosa, or binge-eating disorder; and (iii) ability to provide informed consent and complete self-report questionnaires. The exclusion criteria were any lifetime psychotic disorder, any lifetime bipolar disorder, or any lifetime substance use disorder; severe cognitive impairment; and acute medical instability requiring inpatient treatment. DSM-5 diagnoses were established by experienced clinicians specialised in eating disorders through clinical interview and review of medical records.

### 2.3. Measures

Moral Orientation Guilt Scale (MOGS). The MOGS is a 17-item self-report scale that assesses guilt across four moral-orientation facets: Moral Norm Violation (MNV), Moral Dirtiness (MODI), Empathy, and Harm [25]. It was developed and validated in a large subclinical sample and showed good reliability and construct/convergent validity [25]; it was also used in our pilot ED sample [15], although formal validation specifically in ED populations remains limited. To illustrate the domains, short translated sample items include “I feel guilty if I do not respect figures who, for me, represent authority” (MNV), “When I think about my mistakes, I feel like a bad person” (MODI), “When I see someone suffering, I feel pain for them” (Empathy), and “I feel guilty if my actions or inactions have caused harm to someone” (Harm). Within the MOGS, the Empathy facet specifically captures empathy-related guilt—that is, guilt elicited by the perception of harm, suffering, or burden caused to others—and is therefore conceptually distinct from the broader cognitive–affective construct of general empathy. Composite indices of deontological guilt (MNV + MODI) and altruistic guilt (Empathy + Harm) were computed following the original scoring procedures.

Eating Disorder Inventory 2 (EDI-2). The EDI-2 is a widely used self-report questionnaire that assesses symptoms of eating disorders and related psychological traits [26]. It has been validated in ED samples, including evidence of discrimination among ED, psychiatric outpatient, and community samples [27], and acceptable psychometric properties in treatment-seeking BED and BN samples [28]. All 11 standard EDI-2 subscales were administered. Example item content includes fear of weight gain (EDI-DT; e.g., “I am terrified of gaining weight”) and binge-eating experiences (EDI-B; e.g., “I have gone on eating binges where I felt that I could not stop”). For the prespecified primary hypotheses, the focus was on Bulimia (EDI-B), Asceticism (EDI-A), and Interpersonal Distrust (EDI-ID). Drive for Thinness (EDI-DT) and Body Dissatisfaction (EDI-BD) were included as core symptom indices, and Impulse Regulation (EDI-IR) was included given its relevance to binge–purge behaviours. Exploratory analyses also examined associations with the other EDI-2 subscales: Ineffectiveness (EDI-INEF), Perfectionism (EDI-PERF), Interoceptive Awareness (EDI-IA), Maturity Fears (EDI-MF), and Social Insecurity (EDI-SI). See Supplementary Table S2 for details. 

Demographic and clinical variables. Age, sex, and body mass index (BMI) were recorded at assessment.

### 2.4. Statistical Analysis

The reporting and analytic decisions follow the STROBE guidance for observational studies [29]. Data were screened for missingness and plausibility; no missing questionnaire data were present.

Descriptive statistics are reported as mean ± SD. Because questionnaire subscales are ordinal/Likert-derived and several variables showed non-normal distributions, between-diagnosis differences in guilt facets were tested using Kruskal–Wallis analyses (two-tailed), with epsilon-squared reported as a non-parametric effect size. When omnibus tests were significant, pairwise Mann–Whitney U tests with Holm adjustment were computed. Sensitivity analyses for between-diagnosis differences in guilt indices were additionally performed using ordinary least squares regression models with diagnosis as predictor, adjusting for age, BMI, and sex (HC3 robust standard errors). Full adjusted coefficients are reported in Supplementary Table S1.

Associations between guilt facets and ED psychopathology were examined using Spearman’s rho (two-tailed) between all MOGS indices (MNV, MODI, Empathy, Harm, deontological guilt, altruistic guilt) and all EDI-2 subscales described above. To reduce the risk of false positives due to multiple testing, we prespecified three a priori correlations (primary hypotheses) informed by moral orientation theory and our pilot study [15]: (i) Moral Norm Violation (MNV) with EDI-2 Bulimia (EDI-B), (ii) Empathy with EDI-2 Asceticism (EDI-A), and (iii) altruistic guilt with EDI-2 Interpersonal Distrust (EDI-ID). Holm correction was applied to these three prespecified primary correlations as confirmatory tests, while the Benjamini–Hochberg false discovery rate (FDR) procedure [30] was applied to all remaining correlations, which were treated as exploratory and interpreted cautiously.

To further probe the prespecified Empathy–Asceticism hypothesis, an ordinary least squares regression model was estimated with EDI-2 Asceticism as the dependent variable. To minimise the risk of overfitting and unstable parameter estimates in a modest sample, predictors were limited a priori to sex and the MOGS Empathy facet, which were entered simultaneously; no forward or stepwise variable selection was used [31]. A sensitivity model additionally adjusting for age, BMI, and diagnosis (HC3 robust standard errors) was also estimated.

Analyses were performed in JASP (Version 0.18.3; JASP Team, University of Amsterdam, Nieuwe Achtergracht 129B, Amsterdam, The Netherlands) [32] and independently cross-checked in Python (Version 3.11.2; Python Software Foundation, Beaverton, OR, USA), using SciPy (Version 1.14.1; SciPy Developers) and statsmodels (Version 0.14.3; statsmodels developers).

## 3. Results

### 3.1. Sample Characteristics

Descriptive characteristics are reported in Table 1. Mean age differed across diagnoses, with BED participants being older on average (H(2) = 10.83, *p* = 0.004), and BMI showed the expected diagnostic pattern, being lowest in AN and highest in BED (H(2) = 78.56, *p* < 0.001). The sex distribution did not differ significantly across diagnoses (χ^2^(2) = 1.91, *p* = 0.385).

### 3.2. Guilt Facets by Diagnosis

**Figure 1:**
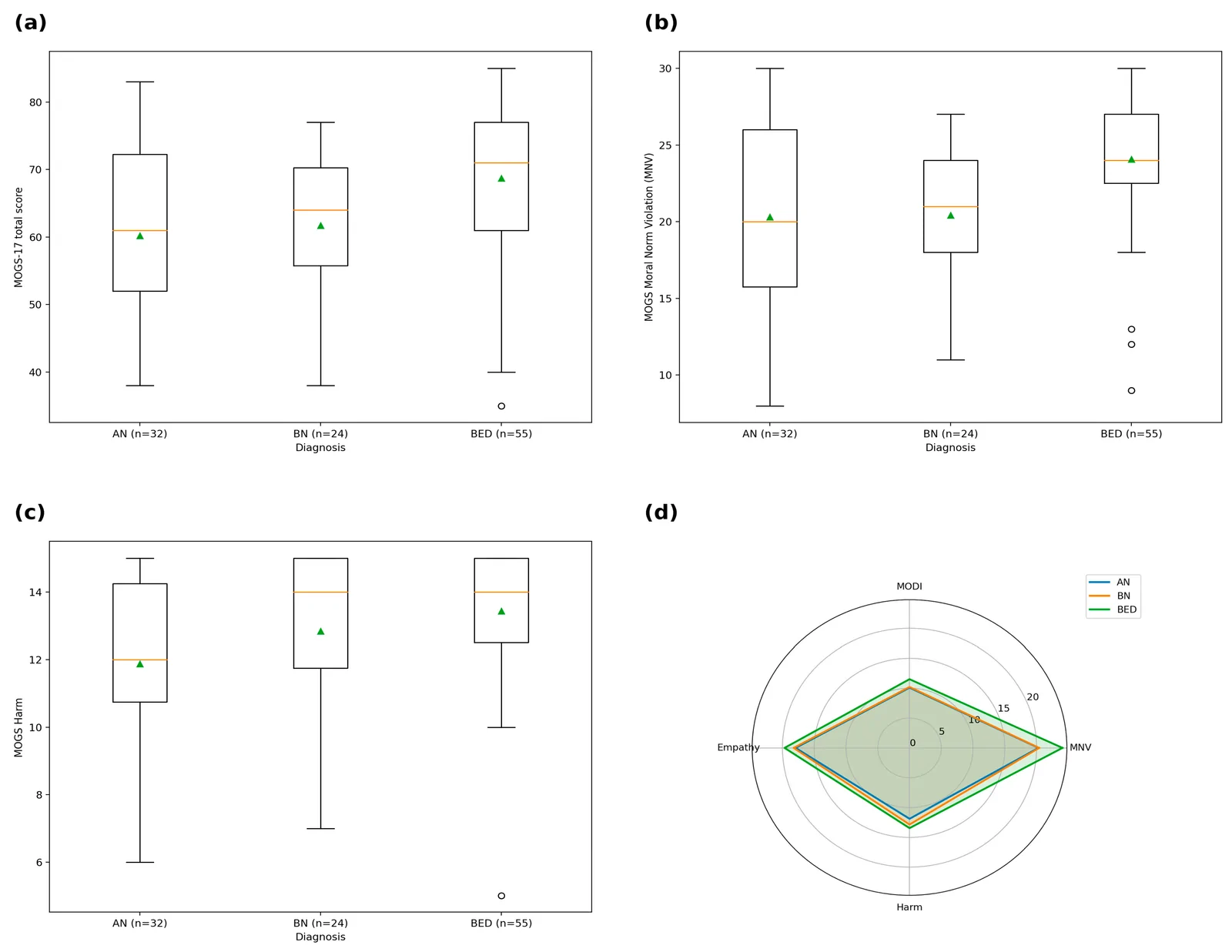
Distribution and profile of MOGS scores by diagnosis. (**a**) MOGS-17 total score; (**b**) Moral Norm Violation (MNV) facet; (**c**) Harm facet; (**d**) mean MOGS facet profile by diagnosis. Abbreviations: AN, anorexia nervosa; BED, binge-eating disorder; BN, bulimia nervosa; MNV, moral norm violation; MOGS, Moral Orientation Guilt Scale.

Mean MOGS scores by diagnosis are shown in Table 2. Distributions of the MOGS total score and selected facets are shown in Figure 1 (Panels a–d). BED showed the highest levels across most guilt facets, followed by BN and AN. Between-diagnosis differences were statistically significant for MNV, MODI, Harm, and both composite indices, whereas Empathy did not differ significantly across diagnoses. Pairwise Mann–Whitney U tests with Holm correction (reported in Table 2) indicated that BED scored higher than both AN and BN on MNV and deontological guilt, higher than AN on Harm and altruistic guilt, and higher than BN on MODI. In sensitivity analyses adjusting for age, BMI, and sex (HC3 robust standard errors), BED remained significantly higher than AN on MNV (B = 3.73, *p* = 0.019), MODI (B = 2.29, *p* = 0.032), Harm (B = 1.45, *p* = 0.044), and deontological guilt (B = 6.02, *p* = 0.011), whereas the BED–AN difference on altruistic guilt did not reach significance after adjustment (B = 2.66, *p* = 0.132). Full adjusted coefficients are reported in Supplementary Table S1.

The between-diagnosis differences are summarised in Table 3.

### 3.3. Associations Between Guilt Facets and ED Psychopathology

**Figure 2:**
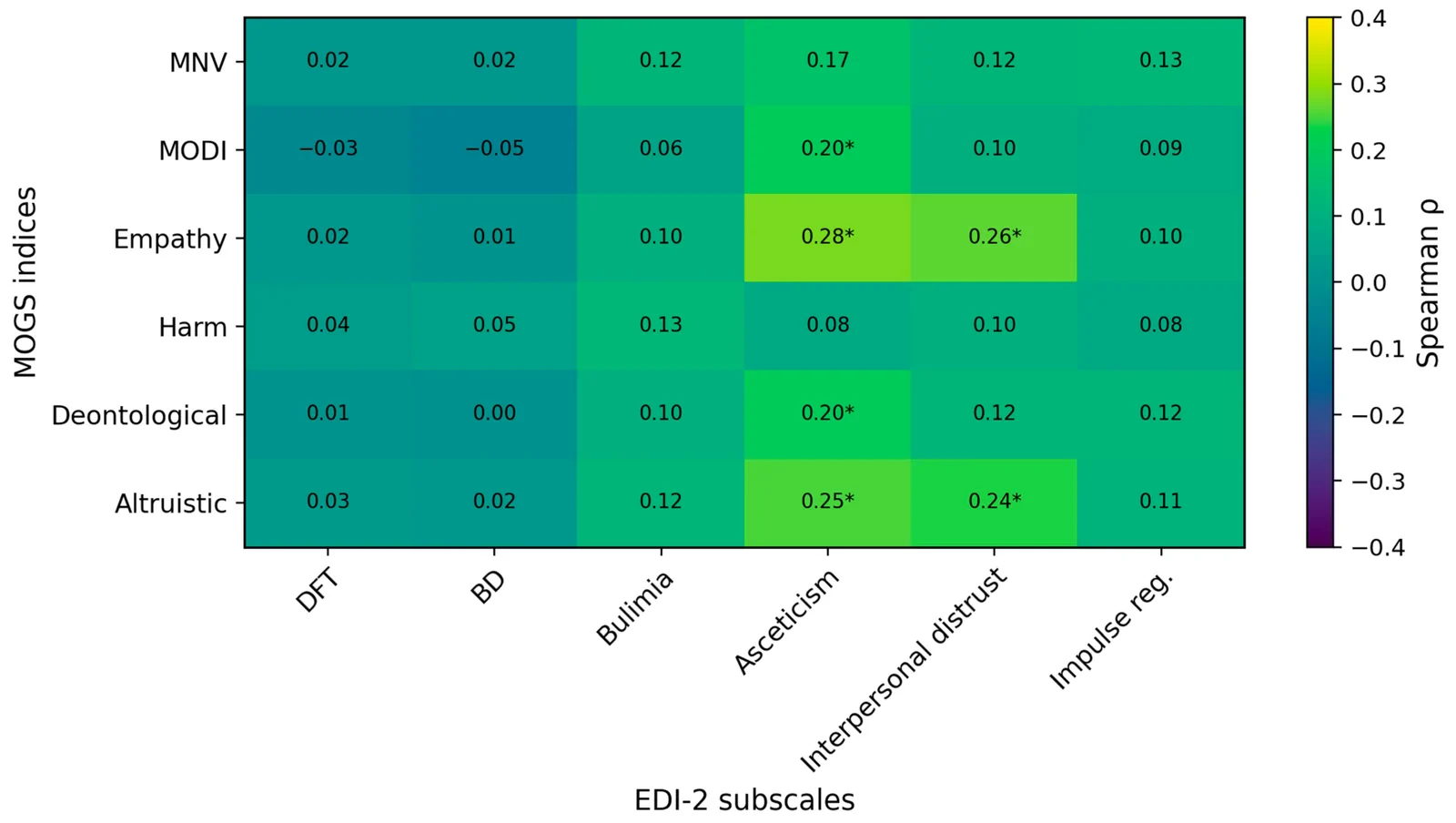
Heatmap of Spearman correlations (ρ) between MOGS indices and selected EDI-2 subscales. Asterisks indicate unadjusted *p* < 0.05 (two-tailed). Abbreviations: EDI-2, Eating Disorder Inventory 2; EDI-A, asceticism; EDI-B, bulimia; EDI-BD, body dissatisfaction; EDI-DT, drive for thinness; EDI-ID, interpersonal distrust; EDI-IR, impulse regulation; MODI, moral dirtiness; MNV, moral norm violation; MOGS, Moral Orientation Guilt Scale.

The correlations between the MOGS indices and the EDI-2 symptom and psychological trait subscales are reported in Supplementary Table S2. Figure 2 shows an overview of the correlation pattern for the selected EDI-2 subscales. Consistent with the prespecified hypotheses, Empathy was positively associated with Asceticism (ρ = 0.28, unadjusted *p* = 0.003; Holm-adjusted *p* = 0.008), while altruistic guilt was positively associated with Interpersonal Distrust (ρ = 0.24, unadjusted *p* = 0.010; Holm-adjusted *p* = 0.020). However, the prespecified association between MNV and Bulimia was not confirmed (ρ = 0.12, unadjusted *p* = 0.199; Holm-adjusted *p* = 0.199). No significant associations were found between guilt indices and EDI-DT, EDI-BD, or EDI-B symptoms in the full sample. Of the EDI-2 psychological traits, Asceticism and Interpersonal Distrust exhibited the strongest positive correlations with guilt indices (see Supplementary Table S2). Beyond the prespecified tests, small positive correlations were observed between Asceticism and MODI, as well as between Asceticism and deontological guilt (both ρ = 0.20, unadjusted *p* = 0.038). However, these did not survive FDR adjustment. Across the broader exploratory correlation matrix, no association survived FDR correction. Figure 2 shows an overview of the correlation pattern.

### 3.4. Exploratory Regression Predicting Asceticism

**Figure 3:**
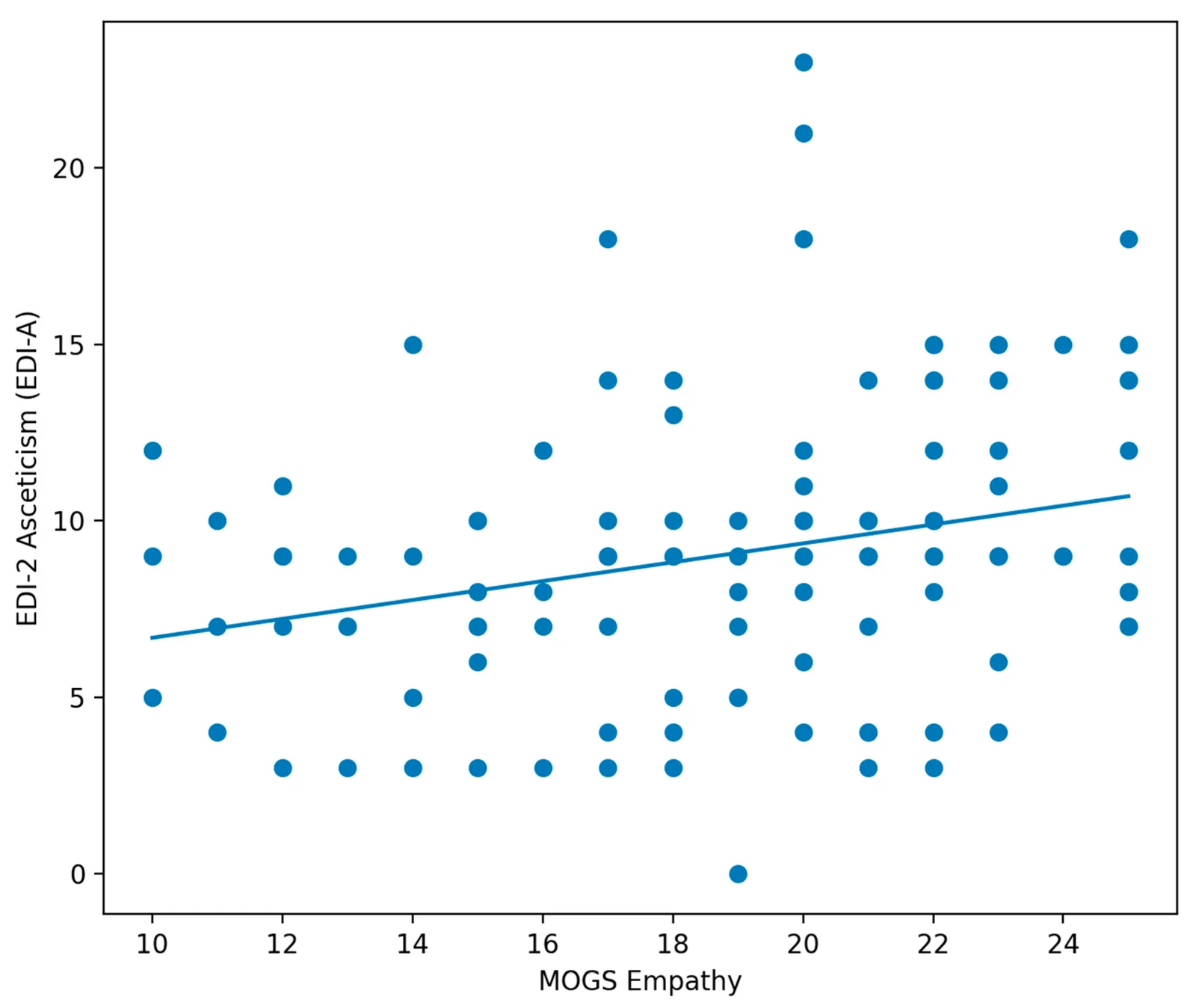
Association between MOGS Empathy and EDI-2 Asceticism (EDI-A). The line represents the fitted linear regression. Abbreviations: EDI-2, Eating Disorder Inventory 2; EDI-A, asceticism; MOGS, Moral Orientation Guilt Scale.

In a regression model testing the association between EDI-2 Asceticism and MOGS Empathy after accounting for sex, Empathy was positively associated with Asceticism (B = 0.263, SE = 0.093, 95% CI 0.078–0.447; standardised β = 0.26; *p* = 0.006). The model explained approximately 7% of the variance in Asceticism (R^2^ = 0.07). In a sensitivity model that adjusted for age, BMI, and diagnosis (HC3 robust standard errors), the Empathy–Asceticism association remained statistically significant (Table 4). The Empathy–Asceticism association is visualised in Figure 3.

## 4. Discussion

This exploratory study examined moral orientation guilt facets in relation to ED diagnosis and ED psychopathology in an adult outpatient sample. Overall, the findings suggest that guilt is not a monolithic construct in eating disorder presentations; rather, its different facets exhibit partially distinct associations with symptomatology.

Diagnosis-related differences were most pronounced for moral norm violation and the deontological guilt composite, with BED showing higher guilt levels than AN (and, for some indices, BN). The pattern is consistent with evidence that guilt is a salient affective state in BED and fluctuates around binge episodes [19]. However, BED participants were also older and had a substantially higher BMI than AN and BN participants (Table 1) so these group differences should not be interpreted as diagnosis-specific without caution. An alternative or additional explanation is internalised weight stigma, whereby people with higher body weight internalise negative weight stereotypes and self-blame; this may amplify weight-related shame/guilt and moralised self-evaluation [22,23,33].

At symptom level, guilt facets were not significantly associated with core ED symptom subscales (Drive for Thinness, Body Dissatisfaction, and Bulimia) in the full sample. This null pattern contrasts with EMA findings showing sharp within-person increases in guilt around binge/purge events [16,17] and close coupling of negative affect (including guilt) with bulimic behaviours over time [20]. One possibility is that trait-like facets of moral orientation guilt (as assessed by the MOGS) more strongly capture motivational and interpersonal processes (e.g., empathic concern and perceived harm) than state-related fluctuations in guilt occurring proximal to specific eating episodes. Another possibility is that diagnosis, BMI, or age-related heterogeneity attenuates cross-sectional associations with symptoms.

The clearest association involved empathy-related guilt and Asceticism. Asceticism reflects self-denial and a tendency to imbue restraint with moral value. From a moral-emotional perspective, high empathic guilt may foster self-sacrifice as a reparative strategy (“I should deprive myself to offset perceived harm/burden”) or as a moral balancing act in contexts of perceived unfairness [10,24]. In our regression, Empathy was positively associated with Asceticism in a parsimonious model, although the variance explained was modest; the association was also retained in a sensitivity model adjusting for age, BMI, and diagnosis. The persistence of the Empathy–Asceticism association after adjustment for diagnosis suggests that this link may operate as a transdiagnostic dimension cutting across AN, BN, and BED, rather than reflecting a diagnosis-specific phenomenon. This pattern is consistent with dimensional frameworks emphasising shared affective–motivational mechanisms across ED categories.

Interpersonal Distrust was associated with Empathy and altruistic guilt. Although seemingly counterintuitive, this combination may reflect a pattern in which empathy-related guilt (i.e., over-concern about harm or burden to others, as operationalised in the MOGS) coexists with defensive interpersonal withdrawal; that is, concern about burdening others may coexist with reduced trust and interpersonal distance.

Comparison with our previous pilot study provides a partial internal replication rather than an independent replication, because the 43 participants included in the pilot are also included in the present expanded sample [15]. In the pilot (*n* = 43), deontological facets were associated with binge/purge features and altruistic guilt was associated with Interpersonal Distrust. In the present expanded sample (*n* = 111), altruistic guilt remained positively associated with Interpersonal Distrust, and the Empathy–Asceticism association was observed again and supported by regression. However, the association between MNV and bulimic symptoms was smaller and did not reach statistical significance, suggesting that this effect may be less stable across samples or more sensitive to diagnostic composition and covariate structure. These convergences and divergences underscore the need for larger, preregistered replications and longitudinal designs.

These findings sit alongside a strong evidence base indicating that shame is robustly associated with ED severity [12,13,14]. Because shame, guilt, and self-criticism are often correlated [10,11], it remains unclear whether guilt facets contribute unique variance beyond shame-related processes in EDs—an important question for future multi-emotion studies. Notably, the most consistent associations of MOGS guilt facets in our sample were not with core ED symptoms but with broader psychopathological dimensions—particularly Asceticism and Interpersonal Distrust—which are not specific to ED psychopathology. This pattern suggests that moral orientation guilt may operate on transdiagnostic features potentially contributing to ED maintenance and treatment resistance, rather than on the symptomatic core of EDs. Our data therefore support a cautious, hypothesis-generating role for guilt facets, complementary to the better-established shame literature. These clinical considerations are hypothesis-generating rather than practice-changing. Because help-seeking barriers and limited access to specialist ED services are common [34], any additional assessment or intervention component related to guilt would need to demonstrate incremental utility and low burden. Future research should test whether brief assessment of guilt facets can support individualised case formulation (e.g., distinguishing primarily rule-violation-based versus empathy/harm-based guilt) and whether targeting the associated appraisals within evidence-based treatments improves outcomes. Where guilt is tightly intertwined with self-criticism and low self-compassion, compassion-oriented modules could be a plausible adjunctive focus, although the current evidence remains preliminary and should be tested against established ED treatments [35,36].

Several limitations warrant emphasis. First, cross-sectional data cannot address directionality: guilt may increase risk for ED behaviours, but ED behaviours can also generate guilt in the short term. Second, although larger than that of our pilot, the sample remains moderate in size and diagnostically imbalanced, limiting precision of group differences and the stability of small correlations. Third, although we identified primary hypotheses informed by theory and our pilot, the overall analytic strategy remains exploratory; multiple correlations raise the risk of type I error, and results should be interpreted primarily in terms of effect sizes and replication across datasets. Fourth, all measures were self-reported; key covariates including trauma history, shame, depression, self-criticism, internalised weight stigma, degree of weight suppression, illness duration, anorexia nervosa subtype (restricting versus binge-eating/purging), current psychotropic medication, and gender identity were not available for all participants and therefore could not be analysed, limiting causal and mechanistic inference. Fifth, our regression model was intentionally parsimonious to reduce overfitting risk and explained a modest proportion of the variance; the results require replication and preferably validation using longitudinal or cross-validated designs.

Future work should (i) test guilt facets alongside shame, self-criticism, trauma, and weight stigma; (ii) employ longitudinal and EMA designs to clarify temporal dynamics and within-person processes; (iii) collect illness duration, weight suppression, anorexia nervosa subtype, and current medication data; (iv) consider lived-experience conceptualisations of recovery, volitional stigma, locus of control, self-blame, and perceived recovery responsibility [37,38,39,40]; and (v) record gender identity as distinct from sex and include transgender and gender-diverse (TGD) participants, given the evidence that ED symptomatology and body-related experiences may differ in TGD populations [41]. Given our pilot and current findings, a preregistered multi-site replication with adequate power and prespecified hypotheses would be a pragmatic next step.

## 5. Conclusions

Guilt facets assessed via moral orientation show selective associations with ED psychopathology in an adult outpatient sample. Empathy-related (altruistic) guilt was linked to Asceticism and Interpersonal Distrust and was associated with Asceticism in an exploratory regression model. Diagnosis-related differences were most evident for moral norm violation, moral dirtiness, Harm, and the composite guilt indices, with higher levels observed in individuals with binge-eating disorder. The findings are hypothesis-generating; robust conclusions require replication in larger and prospective studies.

## Figures and Tables

**Table 1:** Baseline demographic and clinical characteristics by diagnosis.

Variable	AN (*n* = 32)	BN (*n* = 24)	BED (*n* = 55)	Test Statistic	*p*
Age (years)	24.84 ± 7.85	23.71 ± 6.27	33.47 ± 15.07	H(2) = 10.83	0.004
BMI (kg/m^2^)	16.86 ± 1.50	21.66 ± 3.17	29.42 ± 6.43	H(2) = 78.56	<0.001
Female sex, *n* (%)	29 (90.6)	19 (79.2)	44 (80.0)	χ^2^(2) = 1.91	0.385
Male sex, *n* (%)	3 (9.4)	5 (20.8)	11 (20.0)		

Note: Age and BMI are reported as mean ± SD. Age and BMI were compared using Kruskal–Wallis tests; sex distribution was compared using Pearson’s χ^2^ test. Sex was recorded as male/female in clinical records; gender identity was not collected. Abbreviations: AN, anorexia nervosa; BN, bulimia nervosa; BED, binge-eating disorder; BMI, body mass index; SD, standard deviation.

**Table 2:** Baseline guilt characteristics (MOGS; mean ± SD).

Scale	AN (*n* = 32)	BN (*n* = 24)	BED (*n* = 55)	Post-Hoc *p*-Value (Holm-Adjusted)	Between-Diagnosis Pattern
MNV	20.31 ± 5.84	20.42 ± 4.39	24.07 ± 4.56	AN–BED 0.004 *; BN–BED 0.003 *	AN = BN < BED
MODI	10.09 ± 3.35	10.21 ± 2.06	11.51 ± 3.10	BN–BED 0.029 *	BN < BED (AN overlaps)
Empathy	17.94 ± 4.02	18.25 ± 4.15	19.67 ± 4.06	—	AN = BN = BED
Harm	11.88 ± 2.57	12.83 ± 2.57	13.44 ± 1.89	AN–BED 0.013 *	AN < BED (BN overlaps)
Deontological guilt (MNV + MODI)	30.41 ± 8.47	30.62 ± 5.91	35.58 ± 7.06	AN–BED 0.007 *; BN–BED 0.007 *	AN = BN < BED
Altruistic guilt (Empathy + Harm)	29.81 ± 5.90	31.08 ± 6.34	33.11 ± 5.25	AN–BED 0.036 *	AN < BED (BN overlaps)

Note: Post-hoc values are Holm-adjusted *p*-values from pairwise Mann–Whitney U tests; * indicates *p* < 0.05. Between-diagnosis patterns are derived from Holm-adjusted post-hoc tests; ‘overlaps’ indicates a group that does not differ significantly from either of the other two groups. Abbreviations: AN, anorexia nervosa; BN, bulimia nervosa; BED, binge-eating disorder; MODI, moral dirtiness; MNV, moral norm violation; MOGS, Moral Orientation Guilt Scale.

**Table 3:** Between-diagnosis differences in MOGS indices (Kruskal–Wallis tests).

Scale	H (df = 2)	*p* (Two-Tailed)	Epsilon^2^
MNV (AN = BN < BED)	15.44	<0.001	0.12
MODI (BN < BED)	7.84	0.020	0.05
Empathy	4.34	0.114	0.02
Harm (AN < BED)	7.70	0.021	0.05
Deontological guilt (MNV + MODI; AN = BN < BED)	13.50	0.001	0.11
Altruistic guilt (Empathy + Harm; AN < BED)	6.41	0.041	0.04

Note: Significant omnibus tests were followed by pairwise Mann–Whitney U tests with Holm correction. Significant post-hoc patterns are summarised in parentheses in the Scale column; post-hoc values are reported in Table 2. Epsilon^2^ indicates the epsilon-squared effect size.

**Table 4:** Regression models testing the association between MOGS Empathy and EDI-2 Asceticism (EDI-A).

Model	Predictor	B	SE	β	Statistic	*p*	95% CI
Primary	Male	−0.537	1.012	−0.05	t = −0.53	0.597	−2.542 to 1.468
Primary	Empathy (MOGS)	0.263	0.093	0.26	t = 2.82	0.006	0.078 to 0.447
Sensitivity	Male	−0.948	1.211	−0.09	z = −0.78	0.433	−3.322 to 1.425
Sensitivity	Empathy (MOGS)	0.198	0.089	0.20	z = 2.22	0.026	0.023 to 0.372
Sensitivity	Age	0.017	0.038	0.05	z = 0.46	0.646	−0.057 to 0.092
Sensitivity	BMI	0.010	0.097	0.02	z = 0.10	0.922	−0.180 to 0.199
Sensitivity	BN (vs. AN)	1.750	1.050	0.18	z = 1.67	0.096	−0.308 to 3.809
Sensitivity	BED (vs. AN)	2.501	1.523	0.31	z = 1.64	0.101	−0.485 to 5.487

Note: The primary model included sex and MOGS Empathy. The sensitivity model additionally adjusted for age, BMI, and diagnosis using HC3 robust standard errors. AN is the reference category for diagnosis; Male is coded 1 = male, 0 = female. Intercepts are omitted for readability. Primary model R^2^ = 0.07; sensitivity model R^2^ = 0.15. Abbreviations: AN, anorexia nervosa; BED, binge-eating disorder; BMI, body mass index; BN, bulimia nervosa; CI, confidence interval; EDI-2, Eating Disorder Inventory 2; EDI-A, asceticism; HC3, heteroscedasticity-consistent standard error estimator 3; MOGS, Moral Orientation Guilt Scale; SE, standard error.

## Data Availability

Data are available from the corresponding author upon reasonable request (subject to ethical and privacy constraints).

## Supplementary Materials

The following supporting information can be downloaded at https://www.mdpi.com/article/10.3390/medicina62091719/s1 Table S1: Sensitivity models examining diagnosis effects on guilt indices after adjusting for age, BMI, and sex (HC3 robust standard errors); Table S2: Spearman correlations between MOGS indices and EDI-2 subscales (N = 111), including two-tailed *p*-values, FDR-adjusted *p*-values, and Holm-adjusted *p*-values for the three prespecified primary correlations.

## Abbreviations

AN, anorexia nervosa; BED, binge-eating disorder; BMI, body mass index; BN, bulimia nervosa; CBT-E, enhanced cognitive behavioural therapy; CI, confidence interval; DSM-5, Diagnostic and Statistical Manual of Mental Disorders, 5th edition; ED, eating disorder; EDI-2, Eating Disorder Inventory 2; EDI-A, asceticism; EDI-B, bulimia; EDI-BD, body dissatisfaction; EDI-DT, drive for thinness; EDI-IA, interoceptive awareness; EDI-ID, interpersonal distrust; EDI-INEF, ineffectiveness; EDI-IR, impulse regulation; EDI-MF, maturity fears; EDI-PERF, perfectionism; EDI-SI, social insecurity; EMA, ecological momentary assessment; FDR, false discovery rate; HC3, heteroscedasticity-consistent standard error estimator 3; MODI, moral dirtiness; MNV, moral norm violation; MOGS, Moral Orientation Guilt Scale; SD, standard deviation; SE, standard error; TGD, transgender and gender-diverse.
