# Supplementary material for: Guilt Facets and Eating Disorder Psychopathology: An Exploratory Cross-Sectional Study Using the Moral Orientation Guilt Scale
Source: Medicina (Kaunas). 2026 Sep 7;62(9):1719. doi: 10.3390/medicina62091719 (PMC13609088; doi:10.3390/medicina62091719)
Supplement: Supplementary file 1 [file medicina-62-01719-s001.zip › medicina-4436954-supplementary.pdf]

Supplementary Material

Guilt Facets and Eating Disorder Psychopathology: An Exploratory Cross-Sectional Study Using the Moral Orientation Guilt Scale

Supplementary Tables

Table S1. Sensitivity models examining diagnosis effects on guilt indices adjusting for age, BMI, and sex (HC3 robust standard errors).

Outcome	BN vs AN (B)	BN vs AN (p)	BED vs AN (B)	BED vs AN (p)	Age (B)	Age (p)	BMI (B)	BMI (p)	Male (B)	Male (p)	R^2
MNV	0.042	0.977	3.729	0.019	-0.008	0.807	0.007	0.950	0.176	0.873	0.128
MODI	0.432	0.591	2.286	0.032	-0.015	0.571	-0.053	0.459	-0.702	0.338	0.069
EMPATHY	0.463	0.695	1.212	0.342	0.060	0.030	0.011	0.892	-1.164	0.218	0.082
HARM	0.980	0.193	1.449	0.044	0.019	0.273	-0.007	0.897	0.280	0.649	0.093
Deont_Guilt	0.474	0.816	6.015	0.011	-0.023	0.684	-0.046	0.779	-0.526	0.726	0.114
Altr_Guilt	1.443	0.415	2.661	0.132	0.079	0.056	0.004	0.973	-0.885	0.525	0.092

Note: AN is the reference category for diagnosis. Male is coded 1 = male, 0 = female. Abbreviations: BMI, body mass index; MOGS, Moral Orientation Guilt Scale; MNV, moral norm violation; MODI, moral dirtiness.

Table S2. Spearman correlations between MOGS indices and EDI-2 subscales (N = 111). Two-tailed p-values are reported. The three prespecified primary correlations (MNV × EDI-B, Empathy × EDI-A, altruistic guilt × EDI-ID) were Holm-corrected (see also footnote); all remaining correlations are reported with Benjamini–Hochberg FDR-adjusted p-values.

EDI-2 subscale	MOGS index	ρ	p (two-tailed)	p (FDR-adjusted)
Drive for Thinness (EDI-DT)	MNV	0.02	0.811	0.974
Drive for Thinness (EDI-DT)	MODI	-0.03	0.735	0.974
Drive for Thinness (EDI-DT)	EMPATHY	0.02	0.804	0.974
Drive for Thinness (EDI-DT)	HARM	0.04	0.702	0.974
Drive for Thinness (EDI-DT)	Deontological guilt (MNV+MODI)	0.01	0.889	0.978
Drive for Thinness (EDI-DT)	Altruistic guilt (EMPATHY+HARM)	0.03	0.745	0.974
Body Dissatisfaction (EDI-BD)	MNV	0.03	0.798	0.974
Body Dissatisfaction (EDI-BD)	MODI	-0.05	0.600	0.922

Body Dissatisfaction (EDI-BD)	EMPATHY	0.01	0.942	0.991
Body Dissatisfaction (EDI-BD)	HARM	0.05	0.626	0.939
Body Dissatisfaction (EDI-BD)	Deontological guilt (MNV+MODI)	0.00	0.972	0.996
Body Dissatisfaction (EDI-BD)	Altruistic guilt (EMPATHY+HARM)	0.02	0.839	0.978
Bulimia (EDI-B)	MNV	0.12	0.199	0.817
Bulimia (EDI-B)	MODI	0.06	0.536	0.903
Bulimia (EDI-B)	EMPATHY	0.10	0.309	0.817
Bulimia (EDI-B)	HARM	0.13	0.166	0.817
Bulimia (EDI-B)	Deontological guilt (MNV+MODI)	0.10	0.291	0.817
Bulimia (EDI-B)	Altruistic guilt (EMPATHY+HARM)	0.12	0.211	0.817
Impulse Regulation (EDI-IR)	MNV	0.13	0.165	0.817
Impulse Regulation (EDI-IR)	MODI	0.10	0.322	0.817
Impulse Regulation (EDI-IR)	EMPATHY	0.10	0.297	0.817
Impulse Regulation (EDI-IR)	HARM	0.08	0.404	0.846
Impulse Regulation (EDI-IR)	Deontological guilt (MNV+MODI)	0.12	0.218	0.817
Impulse Regulation (EDI-IR)	Altruistic guilt (EMPATHY+HARM)	0.11	0.255	0.817
Interpersonal Distrust (EDI-ID)	MNV	0.12	0.229	0.817
Interpersonal Distrust (EDI-ID)	MODI	0.10	0.319	0.817
Interpersonal Distrust (EDI-ID)	EMPATHY	0.26	0.007	0.163
Interpersonal Distrust (EDI-ID)	HARM	0.10	0.275	0.817
Interpersonal Distrust (EDI-ID)	Deontological guilt (MNV+MODI)	0.12	0.202	0.817
Interpersonal Distrust (EDI-ID)	Altruistic guilt (EMPATHY+HARM)	0.24	0.010	0.163
Asceticism (EDI-A)	MNV	0.17	0.074	0.694
Asceticism (EDI-A)	MODI	0.20	0.038	0.422
Asceticism (EDI-A)	EMPATHY	0.28	0.003	0.163
Asceticism (EDI-A)	HARM	0.08	0.393	0.846
Asceticism (EDI-A)	Deontological guilt (MNV+MODI)	0.20	0.038	0.422
Asceticism (EDI-A)	Altruistic guilt (EMPATHY+HARM)	0.24	0.010	0.163
Ineffectiveness (EDI-INEF)	MNV	-0.02	0.856	0.978

Ineffectiveness (EDI-INEF)	MODI	-0.07	0.476	0.873
Ineffectiveness (EDI-INEF)	EMPATHY	-0.08	0.386	0.846
Ineffectiveness (EDI-INEF)	HARM	-0.04	0.664	0.973
Ineffectiveness (EDI-INEF)	Deontological guilt (MNV+MODI)	-0.03	0.759	0.974
Ineffectiveness (EDI-INEF)	Altruistic guilt (EMPATHY+HARM)	-0.07	0.436	0.846
Maturity Fears (EDI-MF)	MNV	0.06	0.516	0.903
Maturity Fears (EDI-MF)	MODI	0.00	0.996	0.996
Maturity Fears (EDI-MF)	EMPATHY	-0.12	0.211	0.817
Maturity Fears (EDI-MF)	HARM	-0.14	0.147	0.817
Maturity Fears (EDI-MF)	Deontological guilt (MNV+MODI)	0.03	0.730	0.974
Maturity Fears (EDI-MF)	Altruistic guilt (EMPATHY+HARM)	-0.13	0.169	0.817
Social Insecurity (EDI-SI)	MNV	0.13	0.164	0.817
Social Insecurity (EDI-SI)	MODI	0.06	0.562	0.905
Social Insecurity (EDI-SI)	EMPATHY	0.09	0.337	0.825
Social Insecurity (EDI-SI)	HARM	0.01	0.946	0.991
Social Insecurity (EDI-SI)	Deontological guilt (MNV+MODI)	0.11	0.235	0.817
Social Insecurity (EDI-SI)	Altruistic guilt (EMPATHY+HARM)	0.08	0.417	0.846
Perfectionism (EDI-PERF)	MNV	-0.02	0.870	0.978
Perfectionism (EDI-PERF)	MODI	0.03	0.798	0.974
Perfectionism (EDI-PERF)	EMPATHY	0.08	0.430	0.846
Perfectionism (EDI-PERF)	HARM	0.00	0.988	0.996
Perfectionism (EDI-PERF)	Deontological guilt (MNV+MODI)	0.01	0.905	0.979
Perfectionism (EDI-PERF)	Altruistic guilt (EMPATHY+HARM)	0.06	0.528	0.903
Interoceptive Awareness (EDI-IA)	MNV	-0.05	0.589	0.922
Interoceptive Awareness (EDI-IA)	MODI	-0.07	0.465	0.873

Interoceptive Awareness (EDI-IA)	EMPATHY	-0.01	0.880	0.978
Interoceptive Awareness (EDI-IA)	HARM	-0.09	0.352	0.829
Interoceptive Awareness (EDI-IA)	Deontological guilt (MNV+MODI)	-0.06	0.547	0.903
Interoceptive Awareness (EDI-IA)	Altruistic guilt (EMPATHY+HARM)	-0.04	0.681	0.974

Holm-adjusted p-values for the three prespecified primary correlations: MNV × EDI-B, $p = 0.199$; Empathy × EDI-A, $p = 0.008$; altruistic guilt × EDI-ID, $p = 0.020$. Abbreviations: EDI-2, Eating Disorder Inventory–2; FDR, false discovery rate; MNV, moral norm violation; MODI, moral dirtiness; MOGS, Moral Orientation Guilt Scale; ϱ, Spearman correlation coefficient.
